# Associations Between Problematic Internet Use, Attentional Control, and Mental Health Symptoms in Romanian Adults: A Cross-Sectional Study

**DOI:** 10.3390/diseases14060189

**Published:** 2026-05-26

**Authors:** Rebeca-Isabela Molnar, Camelia Sandu, Otilia-Rodica Buțiu, Horia Marchean, Dan Valeriu Nicolae Molnar, Adriana Mihai

**Affiliations:** 1 Doctoral School of Medicine and Pharmacy, George Emil Palade University of Medicine, Science, and Technology of Targu Mures, 540142 Targu Mures, Romania; 2 Psychiatric Clinic 1, Mures County Hospital, 540081 Targu Mures, Romania; 3 Psychiatry Department, George Emil Palade University of Medicine, Science, and Technology of Targu Mures, 540142 Targu Mures, Romania; 4 Neuropsychiatric Clinic, Mures County Hospital, 540005 Targu Mures, Romania; 5 Institute of Psychotherapy and Personal Development, 540038 Targu Mures, Romania

**Keywords:** problematic internet use, attentional control, depression, anxiety, adults, behavioral addiction, Romania, cross-sectional study

## Abstract

Introduction: Problematic internet use has been increasingly associated with depression, anxiety and other psychiatric symptoms; however, its impact on attentional functioning has not been thoroughly researched. This cross-sectional study was conducted in Târgu Mureș, Romania, and aimed to examine the associations between problematic internet use, attentional control, and symptoms of depression and anxiety in adults, and to determine whether problematic internet use independently predicts attentional control after accounting for emotional symptoms. Methods: A cross-sectional study was conducted on 224 adults who completed an anonymous online survey between 1 January 2026 and 1 April 2026. Problematic internet use was assessed using the Compulsive Internet Use Scale-14 (CIUS-14), attentional control using the Attentional Control Scale (ACS), depressive symptoms using the Patient Health Questionnaire-9 (PHQ-9), anxiety symptoms using the Generalized Anxiety Disorder-7 scale (GAD-7), and eating disorder risk using the SCOFF questionnaire. Descriptive statistics, internal consistency analyses, Pearson correlations, group comparisons according to the CIUS-14 screening threshold, and multiple linear regression analyses were performed. Results: Problematic internet use was significantly associated with lower attentional control (r = −0.493, *p* < 0.001), higher depressive symptoms (r = 0.408, *p* < 0.001), and higher anxiety symptoms (r = 0.467, *p* < 0.001). In the regression model, problematic internet use remained the only significant independent predictor of attentional control (B = −0.597, *p* < 0.001), whereas depressive and anxiety symptoms were not significant after adjustment. Participants above the CIUS-14 screening threshold reported significantly lower attentional control and higher depression and anxiety scores than those below the threshold. Conclusions: Problematic internet use was associated with poorer attentional control and greater emotional symptom severity in Romanian adults. These findings suggest that problematic internet use may be linked to a broader cognitive–emotional vulnerability profile. However, because of the cross-sectional design, self-report measures, convenience sampling, and lack of detailed information on specific online activities, the findings should be interpreted cautiously. Longitudinal studies using objective cognitive measures and more detailed assessment of digital behaviors are needed.

## 1. Introduction

The internet is now largely used in daily life and serves many purposes, including communication, work, education, and entertainment. On this note, researchers’ attention has been directed toward patterns of internet use that become excessive, difficult to control, and are associated with functional impairment. As a result, problematic internet use (PIU) has increasingly been discussed as a relevant public health concern, particularly because of its links with impaired self-regulation, emotional distress, and reduced psychosocial functioning [1,2,3]. Although a universally accepted definition and terminology still vary across studies, PIU is generally understood as maladaptive, excessive, or poorly controlled internet use that interferes with everyday functioning [1,2].

Research has shown that PIU is associated with symptoms of depression and anxiety. Previous cross-sectional studies and reviews have consistently reported that individuals with higher levels of problematic internet or smartphone use tend to report greater depressive symptoms, higher anxiety, and a broader burden of psychological distress [4,5,6,7]. Several explanations have been proposed for these hypotheses. Individuals experiencing depression or anxiety may turn to online environments for distraction, escape, reassurance, or emotion regulation. Alternatively, excessive internet use may contribute to maladaptive routines, including sleep disruption, social withdrawal, and overstimulation, which may further intensify emotional symptoms [2,6]. Available evidence suggests that PIU often co-occurs with emotional vulnerability rather than representing an isolated behavioral pattern [7].

Problematic internet use is also a culturally and contextually shaped phenomenon. Patterns of digital engagement may vary according to age, gender, educational background, occupational demands, access to technology, cultural norms regarding online communication, and the role of digital platforms in everyday life. Cross-cultural studies suggest that problematic internet-related behaviors may differ across countries not only in prevalence, but also in the dominant forms of use, such as social networking, gaming, messaging, streaming, or work-related digital engagement. Therefore, examining problematic internet use in Romanian adults may contribute local evidence to a broader international literature and may help clarify whether associations between internet use, emotional symptoms, and attentional control are observable in this specific sociocultural context [8].

In addition to its emotional correlates, PIU has increasingly been examined in relation to cognitive functioning, particularly in domains involving attention and cognitive control. Attention is central to adaptive functioning, as it supports the ability to sustain focus, filter competing information, and shift efficiently between tasks when needed [9,10]. As part of this domain, attentional control refers to the capacity to regulate and direct attention in a goal-oriented manner. This construct is especially relevant in the context of digital environments, which are often characterized by rapid information flow, repeated interruptions, and frequent opportunities for distraction. Existing evidence suggests that problematic forms of internet, smartphone, and social media use may be associated with distractibility, poorer cognitive control, and difficulties in attention regulation [11,12,13,14]. Research on media multitasking and digital distraction has similarly suggested that frequent engagement with highly stimulating digital environments may be linked to reduced attentional stability and less efficient cognitive filtering [15,16,17]. However, this literature remains heterogeneous, as studies differ substantially in terms of age groups, behavioral targets, and the cognitive constructs being assessed.

Beyond emotional symptoms, problematic digital behaviors have increasingly been examined in relation to attention, executive functioning, and broader self-regulatory processes. Previous evidence suggests that problematic internet-related behaviors may be associated with cognitive deficits, including difficulties in inhibitory control, attentional regulation, and executive functioning [11,12,16]. Studies focusing on problematic smartphone or social media use have similarly linked maladaptive digital engagement to attention dysregulation and poorer executive functioning [17,18]. In parallel, work on internet gaming-related behaviors has also suggested a relationship between problematic online engagement and attentional control difficulties [19].

At the same time, the literature remains fragmented. Much of the available evidence has focused on adolescents, university students, or narrower phenotypes such as internet gaming disorder and problematic smartphone use, while fewer studies have examined problematic internet use more broadly in adult populations [4,6,13]. Although cross-sectional and review data suggest that problematic digital behaviors are linked both to emotional symptoms and to attention-related difficulties, these constructs are often studied separately or in partially overlapping populations. This makes it difficult to determine whether attentional control is associated with problematic internet use beyond its shared variance with depression and anxiety, particularly in adults [12,13].

Despite growing interest in this area, several issues remain insufficiently clarified. First, much of the literature has focused on adolescents, university students, gaming disorder, or problematic smartphone use, rather than PIU more broadly in adult populations. Second, although attention-related outcomes are often discussed, fewer studies have specifically examined attentional control as a self-regulatory construct in relation to PIU. Third, because depression and anxiety are themselves associated with both problematic internet use and attentional difficulties, it remains important to determine whether the association between PIU and attentional control persists after accounting for emotional symptoms. This distinction is relevant because it may help clarify whether attentional difficulties reflect only general affective distress or are more specifically linked to maladaptive internet use.

Accordingly, the present study aimed to examine the associations between problematic internet use, attentional control, depressive symptoms, and anxiety symptoms in Romanian adults. Specifically, we investigated whether higher levels of problematic internet use were associated with lower attentional control and greater emotional symptom severity, and whether problematic internet use remained associated with attentional control after accounting for depressive and anxiety symptoms. Given the cross-sectional design, the study was intended to identify associations rather than establish causal relationships.

## 2. Materials and Methods

### 2.1. Study Design

This study used a cross-sectional design to examine the associations between problematic internet use, attentional control, and symptoms of depression and anxiety in adults.

### 2.2. Participants and Recruitment

A total of 224 adults were included in the study. Eligible participants were aged 18 years or older, lived in Romania, and were able to complete a self-administered questionnaire in Romanian. Participants were recruited between 1 January 2026 and 1 April 2026 through online dissemination on social media platforms, as well as through universities and corporate settings. The study was conducted in Târgu Mureș, Romania, using a convenience sampling approach. Participation was voluntary, and responses were collected anonymously. Because recruitment was conducted online and through convenience sampling, the sample should not be considered representative of the general Romanian adult population. This limitation was considered when interpreting the findings.

### 2.3. Procedure

Data were collected using an anonymous online survey. The questionnaire included items on sociodemographic characteristics, problematic internet use, attentional control, depressive symptoms, anxiety symptoms, eating disorder risk, sleep-related aspects, and attitudes toward the credibility of online information. For the purposes of the present study, the main variables of interest were problematic internet use, attentional control, depressive symptoms, and anxiety symptoms.

Before completing the questionnaire, participants were informed about the purpose of the study and the voluntary nature of participation. No personally identifying information was collected.

Prior to analysis, the dataset was examined for duplicate entries, missing data, and response consistency. One duplicate response was identified and removed based on identical values across all substantive variables, with a different submission timestamp. No additional obvious irregularities were observed during data screening. All subsequent analyses were conducted on the cleaned dataset.

### 2.4. Measures

#### 2.4.1. Problematic Internet Use

Problematic internet use was assessed using the Compulsive Internet Use Scale, 14-item version (CIUS-14) [18]. Higher total scores indicate greater problematic internet use. For descriptive purposes, a score of 21 or above was considered indicative of probable problematic internet use.

#### 2.4.2. Attentional Control

Attentional control was assessed using the Attentional Control Scale (ACS), a self-report measure designed to evaluate the individual’s perceived ability to focus and shift attention in goal-directed situations [19]. Higher scores indicate better attentional control.

#### 2.4.3. Depressive Symptoms

Depressive symptoms were assessed using the Patient Health Questionnaire-9 (PHQ-9) [20]. Higher total scores reflect greater depression severity.

#### 2.4.4. Anxiety Symptoms

Anxiety symptoms were assessed using the Generalized Anxiety Disorder-7 scale (GAD-7) [21]. Higher total scores reflect greater anxiety severity.

#### 2.4.5. Eating Disorder Risk

The SCOFF questionnaire was included as a screening measure for eating disorder risk and was analyzed descriptively as part of the broader clinical profile of the sample [22].

### 2.5. Statistical Analysis

Statistical analyses were performed to describe the sample and examine the relationships among the main study variables. Statistical analyses were performed using SPSS version 31.0. Statistical significance was set at *p* < 0.05, using two-tailed tests. Continuous variables were summarized using means and standard deviations, whereas categorical variables were presented as frequencies and percentages.

The internal consistency of the psychometric scales was evaluated using Cronbach’s alpha. Pearson correlation coefficients were calculated to examine associations between problematic internet use, attentional control, depressive symptoms, anxiety symptoms, and SCOFF scores.

To further examine whether problematic internet use was independently associated with attentional control, a multiple linear regression analysis was conducted with ACS total score as the dependent variable and CIUS-14, PHQ-9, and GAD-7 total scores as independent variables. In addition, participants were grouped according to the CIUS-14 screening threshold, and between-group differences in attentional control, depressive symptoms, and anxiety symptoms were explored.

### 2.6. Ethical Considerations

The study was approved by the Ethics Committee of the University of Medicine, Pharmacy, Science and Technology George Emil Palade from Târgu-Mureș (certificate no 3954). All procedures were conducted in accordance with institutional ethical standards and the principles of the Declaration of Helsinki. Participation was voluntary, and all data were collected anonymously.

## 3. Results

### 3.1. Sample Characteristics

A total of 224 adults were included in the analysis. Women represented 51.8% of the sample and men 48.2%. Most participants lived in urban areas (62.5%). The largest age group was 18–25 years (27.7%), followed by 25–35 years (22.8%).

### 3.2. Descriptive Statistics and Reliability

The mean ACS score was 49.47 ± 13.14, the mean CIUS-14 score was 28.66 ± 11.67, the mean PHQ-9 score was 11.26 ± 6.53, and the mean GAD-7 score was 11.57 ± 4.71. Internal consistency was good for ACS (α = 0.866), acceptable for CIUS-14 (α = 0.780) and PHQ-9 (α = 0.793), and lower for GAD-7 (α = 0.679) and SCOFF (α = 0.618) (Table 1).

### 3.3. Clinical Thresholds

Using the commonly applied CIUS-14 screening threshold of ≥21, 170 participants (75.9%) met criteria for probable problematic internet use. On the PHQ-9, 125 participants (55.8%) reported at least moderate depressive symptoms. On the GAD-7, 148 participants (66.1%) reported at least moderate anxiety symptoms (Table 2).

### 3.4. Group Differences According to Probable Problematic Internet Use

Participants meeting the CIUS-14 screening threshold for probable problematic internet use differed significantly from those below the threshold on the main psychological variables. Compared with participants scoring below the threshold, those with probable problematic internet use reported lower attentional control (46.56 vs. 58.63, *p* < 0.001), higher depressive symptoms (12.22 vs. 8.22, *p* < 0.001), and higher anxiety symptoms (12.66 vs. 8.15, *p* < 0.001). This pattern indicates that probable problematic internet use was associated with a more adverse cognitive–emotional profile, characterized by both greater emotional burden and poorer perceived attentional regulation.

### 3.5. Symptom Severity Profile

As shown in Table 2, screening-based severity distributions suggested a substantial emotional burden within the sample. More than half of participants reported at least moderate depressive symptoms, and a considerable proportion also fell within the moderate-to-severe anxiety range. Although these categories should not be interpreted diagnostically, they provide important context for understanding the associations observed between problematic internet use, attentional control, and emotional symptoms.

### 3.6. Correlations Between Problematic Internet Use, Attentional Control, and Mental Health Symptoms

Pearson correlation analyses showed that higher problematic internet use was associated with poorer attentional control, r(222) = −0.493, *p* < 0.001, higher depressive symptoms, r(222) = 0.408, *p* < 0.001, and higher anxiety symptoms, r(222) = 0.467, *p* < 0.001. Depressive and anxiety symptoms were moderately intercorrelated, r(222) = 0.478, *p* < 0.001. The full correlation matrix is shown in Table 3.

### 3.7. Linear Regression Predicting Attentional Control

**Figure 1:**
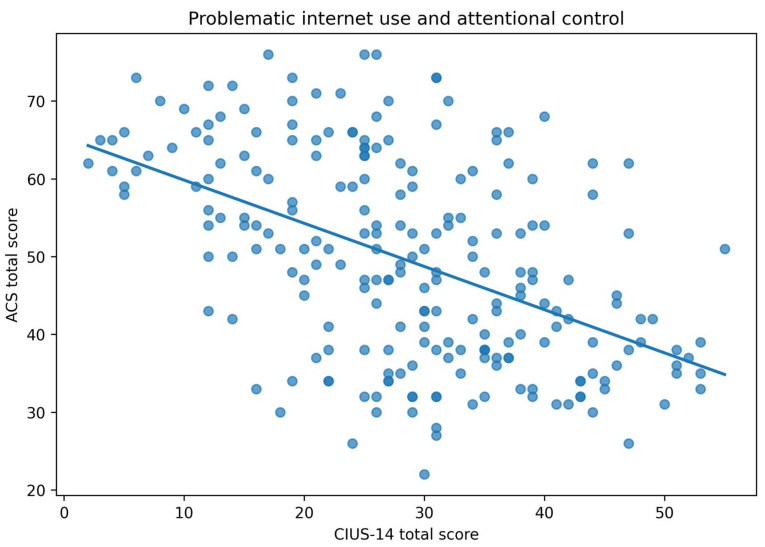
Relationship between problematic internet use and attentional control. Scatterplot showing the inverse association between CIUS-14 and ACS total scores. The fitted line represents the linear trend.

A multiple linear regression model including CIUS-14, PHQ-9, and GAD-7 scores significantly predicted ACS scores, F(3, 220) = 24.10, *p* < 0.001, explaining 24.7% of the variance (adjusted R^2^ = 0.237). CIUS-14 emerged as the only significant independent predictor of attentional control (B = −0.597, SE = 0.077, *p* < 0.001), whereas PHQ-9 and GAD-7 did not remain significant in the adjusted model (Table 4). Collinearity diagnostics did not indicate problematic multicollinearity among predictors, with all VIF values below the commonly used threshold of 5 and tolerance values above 0.20. Figure 1 illustrates the inverse association between problematic internet use and attentional control.

## 4. Discussion

The present study examined the associations between problematic internet use, attentional control, and symptoms of depression and anxiety in adults. Overall, the findings showed a coherent pattern. Higher levels of problematic internet use were associated with lower attentional control and with higher depressive and anxiety symptom severity. In addition, participants above the CIUS-14 screening threshold showed a less favorable cognitive–emotional profile, characterized by lower attentional control and higher levels of depression and anxiety. Importantly, when problematic internet use, depressive symptoms, and anxiety symptoms were considered simultaneously, problematic internet use remained the only significant independent predictor of attentional control. Taken together, these findings suggest that problematic internet use may be linked not only to emotional distress but also to broader difficulties in self-regulatory functioning.

One of the main findings of the study was the inverse association between problematic internet use and attentional control. Participants with higher levels of problematic internet use reported lower ability to focus and shift attention effectively. This result is broadly consistent with previous research suggesting that problematic internet-related behaviors are associated with cognitive and executive difficulties, including impairments in attention regulation, inhibitory control, and broader self-regulatory processes [11,12]. Similar observations have also been reported in studies focusing on problematic smartphone use and problematic social media use, where maladaptive digital engagement has been linked to attention dysregulation and poorer executive functioning [13,14]. In addition, work on internet gaming-related behaviors has suggested a relationship between problematic online engagement and reduced attentional control [15]. The present findings therefore fit within an emerging body of literature indicating that problematic digital behavior may be meaningfully related to everyday attentional regulation.

At the same time, the present study adds to the literature in an important way. Much of the previous research in this area has focused on adolescents, students, or narrower phenotypes such as internet gaming disorder and problematic smartphone use [23,24]. By contrast, the current study examined problematic internet use more broadly in an adult sample and addressed attentional control as a psychologically meaningful self-regulatory construct rather than only as a laboratory-based cognitive outcome. This is relevant because attentional control is closely tied to everyday functioning, including concentration, task persistence, and the ability to resist distraction in academic, occupational, and daily-life settings. In this sense, the study contributes to the literature not simply by replicating an association between digital overuse and cognitive difficulties, but by framing attentional control as a potentially important correlate of problematic internet use in adults [23,25,26].

The study also confirmed that problematic internet use was positively associated with depressive and anxiety symptoms. This finding is in line with a substantial body of prior literature reporting that problematic internet or smartphone use frequently co-occurs with depression, anxiety, and broader psychological distress [9,10,11,12,13]. Similar patterns have been observed across different age groups and digital behavior phenotypes, suggesting that problematic internet-related behaviors often develop within a broader emotional vulnerability context [6]. Several mechanisms may account for these associations. Individuals with depression or anxiety may turn to online environments for distraction, avoidance, reassurance, or emotion regulation, whereas excessive internet use may in turn contribute to maladaptive routines, including sleep disruption, social withdrawal, overstimulation, and reduced offline coping. Although the present design does not allow causal inference, the findings support the view that problematic internet use and emotional distress are closely intertwined.

A particularly noteworthy aspect of the present results is that problematic internet use remained independently associated with attentional control after depressive and anxiety symptoms were entered into the same regression model. This finding is important because it suggests that the relationship between problematic internet use and attentional control was not fully explained by general affective distress. In other words, attentional dysregulation may represent a more specific correlate of problematic internet use rather than merely reflecting the cognitive burden of depression or anxiety. This does not imply causality, nor does it mean that emotional symptoms are secondary or irrelevant. However, it does suggest that problematic internet use may have a distinct relationship with self-regulatory cognitive functioning, which deserves further attention in both research and clinical contexts. In this respect, the present study adds nuance to the existing literature by showing that problematic internet use may be associated with attentional control above and beyond its overlap with depressive and anxiety symptoms [27].

The cultural context of problematic internet use should also be considered. Digital habits are shaped by social norms, educational demands, work routines, access to technology, and preferred online activities. Therefore, the associations observed in Romanian adults may not be directly generalizable to other cultural contexts. Cross-cultural evidence suggests that problematic digital engagement and its mental health correlates may vary across countries and populations, depending on the dominant forms of online activity and sociocultural expectations regarding connectivity. This supports the need for local studies while also encouraging cross-national comparisons.

The group comparisons strengthen this interpretation. Participants meeting the CIUS-14 screening threshold for probable problematic internet use showed lower attentional control and higher depression and anxiety scores than those below the threshold. This pattern suggests that more pronounced problematic internet use is associated with a broader adverse cognitive–emotional profile. From a clinical perspective, this is important because problematic internet use may not present only as excessive screen engagement, but also as part of a cluster of complaints involving distractibility, emotional distress, and reduced self-regulatory capacity. Screening for problematic internet use may therefore be useful in adults presenting with concentration difficulties, anxiety, or depressive symptoms, particularly when digital habits appear difficult to control.

Although no significant association between gender and overall problematic internet use was observed in the present study, this finding should be interpreted cautiously. Gender differences may be more apparent when specific types of digital engagement are examined separately, such as social media use, gaming, messaging, or work-related internet use. Because the present study used a general measure of problematic internet use, it could not determine whether distinct online activities show different gender patterns or different associations with emotional symptoms and attentional control [28].

The present findings may also have broader conceptual implications. They support the idea that problematic internet use should not be understood solely as a lifestyle habit or a simple matter of screen time, but rather as a behavior that may intersect with attention regulation and emotional functioning in clinically meaningful ways. This is relevant for prevention and intervention, as approaches focusing only on reducing time spent online may be insufficient if they do not also address underlying self-regulatory difficulties, emotion regulation needs, or maladaptive coping patterns. Interventions that include psychoeducation, digital self-regulation strategies, attentional management, and broader psychological support may therefore be especially relevant.

Several limitations should be acknowledged. First, the cross-sectional design precludes conclusions regarding causality or temporal direction. It remains unclear whether lower attentional control increases vulnerability to problematic internet use, whether problematic internet use contributes to attentional dysregulation over time, or whether both are shaped by shared underlying factors. Second, all variables were assessed through self-report, which raises the possibility of common method bias and means that attentional control was measured as a perceived rather than an objectively tested construct. Third, the sample was recruited through convenience sampling and may not be representative of the broader Romanian adult population. Fourth, because data were collected through an anonymous online survey, the absence of formal attention-check or infrequent-response items represents a limitation. Although the dataset was screened for duplicates, missing data, and obvious response irregularities, random or careless responding cannot be fully excluded. Fifth, the study assessed problematic internet use globally and did not distinguish between specific online activities such as social media use, gaming, messaging, streaming, work-related internet use, or AI-assisted digital engagement. These activities may have different associations with anxiety, depression, and attentional stability. Sixth, digital competence and confidence in using advanced technologies, including AI-related tools, were not assessed, although technological self-efficacy may influence how individuals experience and report internet-related burden [29]. Finally, ADHD-related traits, impulsivity, inhibitory control, sleep quality, total screen time, and objective cognitive performance were not examined as covariates. Future research should use longitudinal designs and multidimensional assessments that include specific digital behaviors, objective cognitive measures, screen-time indicators, ADHD-related traits, and digital self-efficacy.

Despite these limitations, the study also has several strengths. It addresses a timely and clinically relevant topic, includes an adult sample, and uses established psychometric instruments to examine problematic internet use in relation to both emotional symptoms and attentional control. Importantly, it moves beyond a purely descriptive association between problematic internet use and distress by showing that problematic internet use remained independently associated with attentional control after accounting for depression and anxiety. Overall, the findings support the view that problematic internet use may be linked to a broader cognitive–emotional vulnerability profile and highlight attentional control as a relevant dimension for future research in adult populations.

## 5. Conclusions

The present study found that problematic internet use was associated with lower attentional control and higher levels of depressive and anxiety symptoms in adults. In addition, problematic internet use remained independently associated with attentional control after depressive and anxiety symptoms were taken into account. These findings suggest that problematic internet use may reflect more than a maladaptive behavioral habit alone, as it appears to be linked to a broader pattern of cognitive–emotional vulnerability.

From a clinical perspective, the results highlight the importance of considering problematic internet use in adults who report attentional difficulties or symptoms of depression and anxiety. Screening for problematic internet use may help identify a potentially relevant and modifiable factor in psychological functioning, particularly in individuals whose digital habits have become difficult to regulate.

Future longitudinal studies using behavioral and neurocognitive measures are needed to clarify the direction, mechanisms, and clinical implications of the observed associations.

## Figures and Tables

**Table 1:** Descriptive statistics and internal consistency of study measures.

Measure	*N*	Mean ± SD	Range	Cronbach’s α
Attentional Control Scale (ACS)	224	49.47 ± 13.14	22–76	0.866
Compulsive Internet Use Scale (CIUS-14)	224	28.66 ± 11.67	2–55	0.780
Patient Health Questionnaire (PHQ-9)	224	11.26 ± 6.53	0–27	0.793
Generalized Anxiety Disorder Scale (GAD-7)	224	11.57 ± 4.71	0–21	0.679
SCOFF	224	1.00 ± 1.26	0–5	0.618

**Table 2:** Clinical thresholds and symptom severity distribution.

Indicator	*n*	%
Probable problematic internet use (CIUS-14 ≥ 21)	170	75.9
PHQ-9 minimal (0–4)	35	15.6
PHQ-9 mild (5–9)	64	28.6
PHQ-9 moderate (10–14)	62	27.7
PHQ-9 moderately severe (15–19)	30	13.4
PHQ-9 severe (20–27)	33	14.7
GAD-7 minimal (0–4)	16	7.1
GAD-7 mild (5–9)	60	26.8
GAD-7 moderate (10–14)	93	41.5
GAD-7 severe (15–21)	55	24.6

**Table 3:** Pearson correlations among the main study variables, presented as r (*p*-value).

Variable	ACS	CIUS-14	PHQ-9	GAD-7	SCOFF
ACS	—				
CIUS-14	−0.493 (<0.001)	—			
PHQ-9	−0.149 (0.026)	0.408 (<0.001)	—		
GAD-7	−0.187 (0.005)	0.467 (<0.001)	0.478 (<0.001)	—	
SCOFF	−0.046 (0.498)	−0.028 (0.673)	−0.002 (0.974)	−0.107 (0.109)	—

**Table 4:** Multiple linear regression predicting attentional control (ACS total score).

Predictor	B	SE	T	*p*	95% CI Low	95% CI High
Intercept	64.282	2.344	27.425	<0.001	59.663	68.901
CIUS-14	−0.597	0.077	−7.792	<0.001	−0.749	−0.446
PHQ-9	0.100	0.138	0.726	0.469	−0.172	0.372
GAD-7	0.102	0.198	0.518	0.605	−0.287	0.492

## Data Availability

The data supporting the findings of this study are not publicly available due to ethical and privacy restrictions related to anonymous survey data and participant confidentiality. Data may be made available by the corresponding author upon reasonable request and subject to institutional approval.

## References

[1] Montag C., Wegmann E., Sariyska R., Demetrovics Z., Brand M. (2021). How to overcome taxonomical problems in the study of Internet use disorders and what to do with “smartphone addiction”?. J. Behav. Addict..

[2] Fineberg N.A., Menchón J.M., Hall N., Dell’Osso B., Brand M., Potenza M.N., Chamberlain S.R., Cirnigliaro G., Lochner C., Billieux J. (2022). Advances in problematic usage of the internet research—A narrative review by experts from the European network for problematic usage of the internet. Compr. Psychiatry.

[3] Lopez-Fernandez O., Romo L., Kern L., Rousseau A., Lelonek-Kuleta B., Chwaszcz J., Männikkö N., Rumpf H.-J., Bischof A., Király O. (2023). Problematic internet use among adults: A cross-cultural study in 15 countries. J. Clin. Med..

[4] Kitazawa M., Yoshimura M., Murata M., Sato-Fujimoto Y., Hitokoto H., Mimura M., Tsubota K., Kishimoto T. (2018). Associations between problematic Internet use and psychiatric symptoms among university students in Japan. Psychiatry Clin. Neurosci..

[5] Elhai J.D., Levine J.C., Hall B.J. (2019). The relationship between anxiety symptom severity and problematic smartphone use: A review of the literature and conceptual frameworks. J. Anxiety Disord..

[6] Guo W., Tao Y., Li X., Lin X., Meng Y., Yang X., Wang H., Zhang Y., Tang W., Wang Q. (2020). Associations of Internet Addiction severity with psychopathology, serious mental illness, and suicidality: Large-sample cross-sectional study. J. Med. Internet Res..

[7] Zhou Y., Zhou Y., Zhou J., Shen M., Zhang M. (2022). Attentional biases and daily game craving dynamics: An ecological momentary assessment study. J. Behav. Addict..

[8] Hoque M., Qadri S.M., Qadri A.A., Khan M.U.H., Adedia D., Uzzaman A., Kwasi F. (2026). Nexus Between Social Media Use and Mental Health Outcomes among High School Students in Kashmir, India. Ianna J. Interdiscip. Stud..

[9] Kahneman D. (1973). Attention and Effort.

[10] Posner M.I., Petersen S.E. (1990). The Attention System of the Human Brain. Annu. Rev. Neurosci..

[11] Firth J.A., Torous J., Firth J. (2020). Exploring the impact of internet use on memory and attention processes. Int. J. Environ. Res. Public Health.

[12] Ioannidis K., Hook R., Goudriaan A.E., Vlies S., Fineberg N.A., Grant J.E., Chamberlain S.R. (2019). Cognitive deficits in problematic internet use: Meta-analysis of 40 studies. Br. J. Psychiatry.

[13] Toh W.X., Ng W.Q., Yang H., Yang S. (2021). Disentangling the effects of smartphone screen time, checking frequency, and problematic use on executive function: A structural equation modelling analysis. Curr. Psychol..

[14] Arness D.C., Ollis T. (2022). A mixed-methods study of problematic social media use, attention dysregulation, and social media use motives. Curr. Psychol..

[15] Ophir E., Nass C., Wagner A.D. (2009). Cognitive control in media multitaskers. Proc. Natl. Acad. Sci. USA.

[16] Uncapher M.R., Thieu M.K., Wagner A.D. (2015). Media multitasking and memory: Differences in working memory and long-term memory. Psychon. Bull. Rev..

[17] Wilmer H.H., Sherman L.E., Chein J.M. (2017). Smartphones and cognition: A review of research exploring the links between mobile technology habits and cognitive functioning. Front. Psychol..

[18] Meerkerk G.J., Van Den Eijnden R.J., Vermulst A.A., Garretsen H.F. (2009). The Compulsive Internet Use Scale (CIUS): Some psychometric properties. Cyberpsychology Behav..

[19] Derryberry D., Reed M.A. (2002). Anxiety-related attentional biases and their regulation by attentional control. J. Abnorm. Psychol..

[20] Kroenke K., Spitzer R.L., Williams J.B.W. (2001). The PHQ-9: Validity of a Brief Depression Severity Measure. J. Gen. Intern. Med..

[21] Spitzer R.L., Kroenke K., Williams J.B.W., Löwe B. (2006). A Brief Measure for Assessing Generalized Anxiety Disorder: The GAD-7. Arch. Intern. Med..

[22] Morgan J.F., Reid F., Lacey J.H. (1999). The SCOFF questionnaire: Assessment of a new screening tool for eating disorders. BMJ.

[23] Méndez M.L., Padrón I., Fumero A., Marrero R. (2024). Effects of internet and smartphone addiction on cognitive control in adolescents and young adults: A systematic review of fMRI studies. Neurosci. Biobehav. Rev..

[24] Ding K., Shen Y., Liu Q., Li H. (2023). The effects of digital addiction on brain function and structure of children and adolescents: A scoping review. Healthcare.

[25] Wang Y., Zou Z., Song H., Xu X., Wang H., Uquillas F.D., Huang X. (2016). Altered gray matter volume and white matter integrity in college students with mobile phone dependence. Front. Psychol..

[26] Nikolaidou M., Fraser D.S., Hinvest N. (2019). Attentional bias in Internet users with problematic use of social networking sites. J. Behav. Addict..

[27] Liu D., Liu X., Long Y., Xiang Z., Wu Z., Liu Z., Bian D., Tang S. (2022). Problematic smartphone use is associated with differences in static and dynamic brain functional connectivity in young adults. Front. Neurosci..

[28] Throuvala M.A., Griffiths M.D., Rennoldson M., Kuss D.J. (2020). Mind over matter: Testing the efficacy of an online randomized controlled trial to reduce distraction from smartphone use. Int. J. Environ. Res. Public Health.

[29] Olijo I.I. (2025). Gender Disparities in Research Return Rates: The Moderating Influence of AI Self-Efficacy and Methodological Design. Verlumun J. AI Gend. Cult. Stud..

